# Secondary Hemophagocytic Lymphohistiocytosis: A Challenging Diagnosis in a Patient with Autoimmune Hepatitis

**DOI:** 10.1155/2019/3580796

**Published:** 2019-02-04

**Authors:** Colin Casault, Juan G. Posadas-Calleja

**Affiliations:** Department of Critical Care Medicine, University of Calgary, Alberta, Canada

## Abstract

**Background:**

We describe a case of secondary Hemophagocytic Lymphohistiocytosis (HLH) from autoimmune hepatitis mimicking severe sepsis in a man admitted to the intensive care unit.

**Case Presentation:**

A 34-year-old Pakistani male with a prior history of biopsy-proven autoimmune hepatitis presented to a regional hospital with severe fever, cytopenias, hyperferritinemia, hypertriglyceridemia, splenomegaly, and a bone marrow biopsy showing hemophagocytosis. After ruling out mimicking conditions, a diagnosis of HLH was made using the HLH-2004 diagnostic criteria. He was treated with dexamethasone and etoposide, without bone marrow transplantation (BMT) due to poor functional status. At one-year after follow-up, he had returned to his baseline functional status without recurrence.

**Conclusion:**

We describe a rare case of secondary HLH in the setting of autoimmune hepatitis. Broadly, this case report educates clinicians to consider this potentially missed diagnosis. This case also informs clinicians that treatment of secondary HLH with BMT may not be necessary for the management of secondary HLH due to autoimmune hepatitis. Finally, it provides a detailed description of the natural history of a single patient with secondary HLH due to autoimmune hepatitis.

## 1. Case Presentation

A 34-year-old male with stage IV cirrhosis secondary to autoimmune hepatitis (AH) and concomitant alcoholism presented to a regional hospital emergency room with fever, vomiting, and altered mentation in the setting of presumed alcohol withdrawal. His prior diagnosis of AH was made two years' prior via transhepatic biopsy and treatment was initiated with azathioprine and prednisone; however, the patient was nonadherent. He immigrated from Pakistan in 2009 and was married with a 4-year-old daughter with no family history of autoimmunity.

At the initial assessment, the patient was hyperthermic at 41.5°C, tachycardic with a heart rate of 132 beats per minute, and tachypneic at 24 breaths per minute with normal oxygen saturation. Bedside examination revealed livedo reticularis of his lower extremities with palmar erythema and spider nevi. His abdomen was slightly firm with tenderness in his right upper quadrant. No organomegaly or peritoneal signs were identified.

Due to suspected sepsis and severe alcohol withdrawal, he was transferred to the ICU for intubation and agitation management. Additionally, broad-spectrum antimicrobial treatment was initiated with coverage for spontaneous bacterial peritonitis and presumed community-acquired meningitis with meropenem, vancomycin, and acyclovir. Over the coming four days, his level of consciousness continued to decline and he developed seizures.

## 2. Investigations

**Figure 1:**
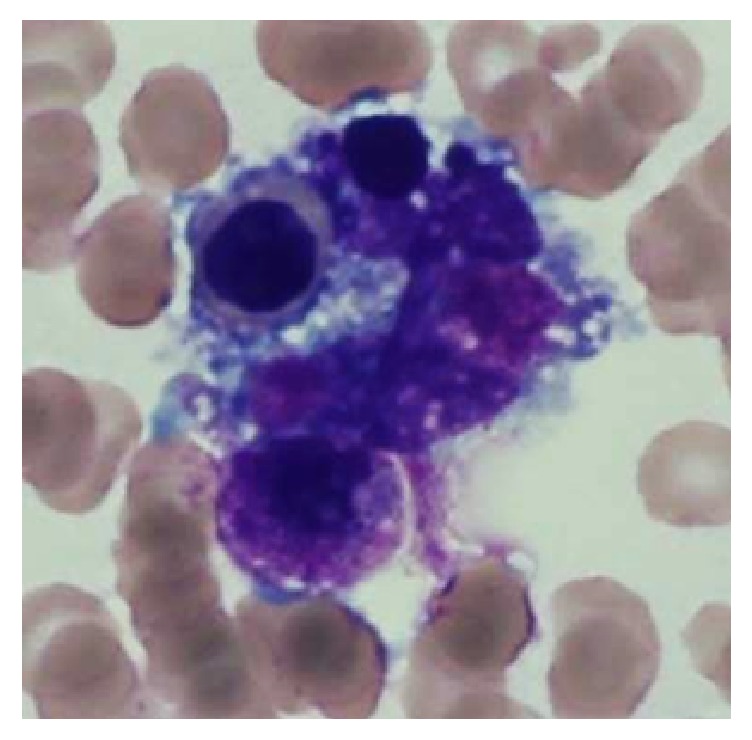
Bone marrow biopsy demonstrating a macrophage containing a late stage erythroid precursor compatible with hemophagocytosis.

Diagnostic workup revealed severe acute thrombocytopenia (9000/mm3) and neutropenia (1100/mm3) compared to normal values a week prior. Blood chemistry demonstrated a mixed cholestatic and hepatocellular enzyme elevation. Over four days, AST values increased rapidly to 2388 IU/L with worsening direct hyperbilirubinemia (40 mcg-mol/L). Synthetic function demonstrated an INR of 1.2, normal glucose and hypoalbuminemia (25g/L). CT imaging of the chest, abdomen and pelvis showed enlargement of the spleen with evidence of hepatic cirrhosis. Ferritin levels were significantly elevated at greater than 8000 mcg/L with an elevated triglyceride level at 10.35 mmol/L. Bone marrow biopsy showed hemophagocytosis and increased macrophage infiltration (see Figure 1).

Infectious workup included extensive blood and urine testing for Hepatitis A, B, C, HIV, herpes simplex, varicella, cytomegalovirus (CMV), Epstein Barr Virus (EBV), acid-fast bacilli, and respiratory viruses. Bone marrow testing for leishmaniasis was negative. Rheumatologic workup yielded a >1:640 speckled ANA pattern, with positive beta-2 microglobulin and lupus type inhibitors present. Extensive workup demonstrated normal C3/4, dsDNA, anti-cardiolipin antibodies, MPO/PR3, anti-smith, AMA, ANCAs, anti-GBM, and HLA phenotyping were all normal. Toxic alcohol screen was similarly negative. Repeat transhepatic biopsy demonstrated known cirrhosis and no evidence of on-going AH. Finally, evaluation for malignancy including enhanced body CT imaging, serum flow cytometry, and immunoglobulins were all normal.

## 3. Management and Clinical Follow-Up

In consultation with Rheumatology and Hematology, his clinical presentation was in keeping with a diagnosis of Hemophagocytic Lymphohistiocytosis (HLH). Treatment was initiated with modified HLH-94 therapy including etoposide and dexamethasone chemotherapy. Intrathecal methotrexate was considered, however, forgone secondary to poor premorbid functional status, severe thrombocytopenia, and significant hepatic dysfunction.

He underwent five months of chemotherapy with clinical response measured in improving hepatic and hematologic parameters. Consolidative bone marrow transplantation was not completed as he was not deemed medically fit under the guidance of hematology. Approximately one-year after diagnosis, he remains in remission with on-going close hematology follow-up.

## 4. Discussion

HLH is a rare clinical syndrome, marked by excessive immune activation causing fever, hepatosplenomegaly, cytopenias, and organ infiltration by activated macrophages, which has an incidence of 1 per 800,000 adults per year [1]. HLH can be categorized into primary and secondary HLH. The former category encapsulates five genetic forms of HLH, which include mutations in Perforin-1, UNC13D, STX11, and STXBP2 proteins [2]. Mutation in the aforementioned proteins leads to impaired natural killer (NK) cell or cytotoxic T-cell activity due to ineffective perforin function or ineffective trafficking, docking and exocytosis of perforin-containing granules. As a result of impaired killing function, NK and cytotoxic T-cells excessively release cytokines and cause immune hyperactivation [2]. Comparatively, secondary HLH represents cases due to a clear trigger which includes both infectious and noninfectious etiologies. Noninfectious triggers may include medications, autoimmune, neoplastic and idiopathic causes. Why a patient develops secondary HLH remains unclear, however experts theorize a “second hit hypothesis” may be important for its development. The “second hit hypothesis” describes a genetically susceptible individual, who requires a sufficient external pressure to manifest the features of HLH [2]. This theory is supported by the discovery of associated genetic polymorphisms involved in granule-mediated cytotoxicity, microtubule organization, vesicular transport, NK-cell receptors, and many other pathways involved in immune regulation [2].

Establishing the correct diagnosis of HLH can be challenging as the clinical manifestations and investigations are akin to septicemia. Fever, encephalopathy, leucopenia, thrombocytopenia, and hypofibrinogenemia may be present in both septic patients and those with HLH. Therefore, systematic evaluation for both infectious and noninfectious contributors remains essential. Viral infections, specifically from the herpes family, including herpes simplex virus (62%), EBV (43%), and CMV (9%), are the most common triggers [1, 3]. Bacterial causes, most often tuberculosis, have been reported in up to 9% [1]. In the immunosuppressed, HLH may be related to opportunistic infection such as toxoplasmosis or fungi [4, 5]. Secondary HLH has also been described as a complication of autoimmune disease, including SLE and Adult-Onset Still's Disease (AOSD) with a prevalence of 4.6% [6]. Most commonly in the setting of autoimmunity, cases of HLH are related to infection due to immunosuppression or malignancy [7, 8]. In the absence of other cause, autoimmune conditions may predispose one to develop HLH due to prolonged stimulation of the innate immune system. The resulting cytokine storm causes functional NK-cell impairment which further contributes to hypercytokinemia [9].

After ruling out other cause, the diagnosis of HLH can be made using the HLH-2004 criteria (Table 1) [1]. In our case, acute onset cytopenias, hyperferritinemia, hypertriglyceridemia, fever, splenomegaly, and a bone marrow biopsy showing hemophagocytosis in the absence of other causes confirmed the diagnosis of HLH. However, the limitations of the HLH-2004 criteria should be acknowledged. They were initially chosen to describe a pediatric population of mainly primary, not secondary HLH. This precedent is reflected in the criteria by the inclusion of molecular markers which are rarely present in adult HLH. Moreover, systemic organ dysfunction may mimic some features of the HLH-2004 diagnostic criteria. For example, hepatic cirrhosis may cause thrombocytopenia, hypofibrinogenemia, and splenomegaly. In our case, the development of acute onset, severe thrombocytopenia, in the context of the supporting clinical features, implies the development of a secondary contributing process rather than splenic sequestration. Clinicians should be cautious about applying the HLH-2004 criteria in the setting of confounding variables. Acknowledging this discrepancy, Fardet et al. developed the h-score, a probability model based on data collected from a multicenter adult cohort with secondary HLH [10]. With a score of >169, the h-score yielded a sensitivity and specificity of 93% and 86%, respectively, for the diagnosis of a hemophagocytic syndrome. Applying this knowledge to our case, our patient's h-score was 264, reflective of a >99% probability of a hemophagocytic syndrome.

Specific to AH, cases are rare, and the natural history remains unclear. Saito et al. report a 15-year-old woman with probable AH and secondary HLH who demonstrated clinical remission after treatment with prednisolone and cyclosporine A [11]. Similarly, Hayashi et al. describe a 60-year-old woman with drug-induced definite AH and secondary HLH who responded to treatment with plasma exchange and immunosuppression with prednisolone [12]. Both cases presented with acute hepatic failure without coma, cytopenias, positive ANA testing, hypergammaglobulinemia, with an absence of fever and met only four HLH-2004 criteria. As such, the diagnosis of AH and HLH were made concurrently. Alternatively, our patient presented with an established diagnosis of AH, based on hepatic biopsy, ANA positivity, and elevated IgG levels, followed two years later by a fulminant presentation with severe fevers, mixed hepatic dysfunction, cytopenias, coma, seizures and met 6 HLH-2004 diagnostic criteria. At that time, normal IgG levels had normalized. All three cases attained remission after immunosuppression with corticosteroids and additional adjuvant treatment. Interestingly, AH has been observed to precede a diagnosis of primary HLH with marked hypogammaglobulinemia and atypical focal brain lesions due to STXBP2 mutation [13]. In comparison to the above cases, this patient died soon after the diagnosis during HLH-2004 induction chemotherapy due to sepsis.

Mortality risk in HLH appears to be dependent on the trigger, which underscores the importance of establishing the diagnosis. Regardless of cause, adult HLH carries a significant mortality risk of 69% with a median overall survival of 4 months. Poor prognostic factors in HLH include age, thrombocytopenia, and etiology [14]. HLH secondary to autoimmunity appears to have a more favorable prognosis with an overall mortality of 13% [15]. All three AH cases with secondary HLH described above demonstrated clinical remission after immunosuppression. This appears supportive that HLH secondary to AH may be more amenable to less intensive treatment regimens than other forms of secondary HLH. More extensive studies are required to evaluate whether patients with AH and HLH carry a more favorable prognosis.

## 5. Conclusion

Differentiating between septicemia and secondary HLH is a diagnostic challenge. Both share many common clinical features including severe fevers, cytopenias, hyperferritinemia, and hypofibrinogenemia with treatment pathways differing dramatically. Utilization of the HLH-2004 diagnostic criteria or other clinical scoring systems, like the h-score, may help identify at-risk patients and multidisciplinary expertise can help secure a diagnosis. Our case provides an excellent example for the challenges of diagnosing secondary HLH as we report a rare patient suffering from secondary HLH from AH.

## Additional Points

*Key Points*. (i) HLH is a rare, life threatening condition with two described forms: Primary and Secondary. (ii) Secondary HLH, primarily seen in adults, is often initiated by an infectious, malignancy, medication, or autoimmune related trigger. (iii) Due to the nonspecific symptoms and high mortality, a high degree of clinical suspicion is required to differentiate between septic shock and HLH. (iv) Knowledge of the HLH-2004 diagnostic criteria or use of a probability score like the h-score reduces the likelihood of missing the diagnosis.

## Figures and Tables

**Table 1:** HLH-2004 diagnostic guidelines[1].

**Criteria**	
Fever	>38.5°C or more
Splenomegaly	Present
Cytopenias	2 cell lines
Hemoglobin	< 90 mmol/L
Thrombocytopenia	< 100 x 10^9^/L
Neutropenia	< 1 x 10^9^/L
Hyperferritinemia	> 1123.5 pmol/L
Hypertriglyceridemia(fasting) and/or hypofibrinogenemia	> 3 mmol/L and/or < 1.7 mmol/L
Hemophagocytosis	Pathologic evidence in the bone marrow, spleen, lymph nodes or liver
Soluble CD25 levels (alpha chain of the soluble interleukin 2 receptor)	Elevated
Natural-killer-cell activity	Low or absent

OR	

Molecular Diagnosis consistent with HLH	Pathological mutations of PRF1, UNC13D, STXBP1, RAB27A, STX11, SH2D1A or XIAP

## Data Availability

Data sharing is not applicable to this article as no datasets were generated or analyzed during the current study.

## Abbreviations

ASOD: Adult-Onset Still's Disease
AH: Autoimmune hepatitis
BMT: Bone marrow transplantation
CMV: Cytomegalovirus
EBV: Epstein Barr Virus
HLH: Hemophagocytic Lymphohistiocytosis
ICU: Intensive Care Unit
NK: Natural Killer.
